# The feasibility and satisfaction study of 5G-based robotic teleultrasound diagnostic system in health check-ups

**DOI:** 10.3389/fpubh.2023.1149964

**Published:** 2023-07-10

**Authors:** Jia-Yu Ren, Yu-Meng Lei, Bing-Song Lei, Yue-Xiang Peng, Xiao-Fang Pan, Hua-Rong Ye, Xin-Wu Cui

**Affiliations:** ^1^Department of Medical Ultrasound, Tongji Hospital, Tongji Medical College, Huazhong University of Science and Technology, Wuhan, China; ^2^Department of Medical Ultrasound, China Resources & Wisco General Hospital, Wuhan University of Science and Technology, Wuhan, China; ^3^Department of Ultrasound, Wuhan Third Hospital, Tongren Hospital of Wuhan University, Wuhan, China; ^4^Health Medical Department, Dalian Municipal Central Hospital, Dalian, China

**Keywords:** robot, teleultrasound, 5G, check-up, underserved areas, telemedicine

## Abstract

**Objective:**

Regular check-up with ultrasound in underserved rural and/or remote areas is hampered due to the limited availability of sonologists and ultrasound devices. This study aimed to assess the feasibility and satisfaction of health check-ups with a 5G-based robotic teleultrasound diagnostic system.

**Methods:**

In this prospective study, sonologists from two hospitals manipulated the telerobotic ultrasound system to perform teleultrasound check-ups of the liver, gallbladder, pancreas, spleen, kidneys, bladder, prostate (male), uterus and ovaries (female) for the subjects. The feasibility and satisfaction of health check-ups with a 5G-based robotic teleultrasound diagnostic system were evaluated in terms of examination results, examination duration, and satisfaction questionnaire survey.

**Results:**

A total of 546 subjects were included with the most frequently diagnosed being abdominal disorders (*n* = 343) and male reproductive illnesses (*n* = 97), of which fatty liver (*n* = 204) and prostatic calcification (*n* = 54) were the most. The median teleultrasound examination duration (interquartile range) for men and women was 9 (9–11) min and 9 (7–11) min (*p* = 0.236), respectively. All the subjects were satisfied with this new type of telerobotic ultrasound check-ups and 96% reported no fear of the robotic arm during the examination.

**Conclusion:**

The 5G-based teleultrasound robotic diagnostic system in health check-ups is feasible and satisfactory, indicating that this teleultrasound robot system may have significant application value in underserved rural and/or remote areas to mitigate disparity in achieving health equity.

## Introduction

Little has changed in traditional physical exam over the last 200 years (1), and it contributes only 10–20% to the final diagnoses (2). In addition, it is impossible for a traditional physical exam to discover diseases without obvious clinical symptoms and signs as only limited exams are performed. A recent study found that an ultrasound screening exam could detect 94% of abnormalities, the great majority of which would have been undetected by a traditional physical exam (3). As a crucial supplement to the traditional hands-on exam, ultrasound is becoming an indispensable third eye and a new figurative stethoscope for clinical physicians due to its affordability, excellent image quality, and functional diversity (4). Regular screening with ultrasound seeks to recognize preclinical cancer and/or precancerous lesions in a seemingly healthy population, which is helpful for cancer early diagnosis and treatment (5).

Although professional medical health services are widely accessible in urban, the availability of regular ultrasound check-ups in a traditional on-site manner is limited in underserved rural and/or remote areas owing to the shortage of skilled sonologists and ultrasound devices (6). It often forces people to travel long distances to a nearby larger hospital for an ultrasound check-up, increasing their economic costs and the larger hospital’s burdens. In some cases, numerous challenges in accessing ultrasound check-ups made them abandon pursuing an ultrasound scan, resulting in a delay in diagnosis and/or therapy and the progress of advanced diseases. Therefore, a creative method is imperative to minimize the inequity in the allocation of health resources and services.

Telemedicine is reckoned as such a unique solution to address this problem. Many studies have indicated that telemedicine can mitigate supply–demand imbalance and improve access to medical imaging services by overcoming geographical barriers, and finally realizing the effective and rational allocation of medical resources (7, 8). Telerobotic ultrasound, as a significant branch of telemedicine, allows sonologists to manipulate the teleultrasound probe to remotely perform real-time ultrasound scanning, making the availability of ultrasound imaging services locally and off-site sonography expertise a reality (9). Previous studies have evaluated the feasibility of applying a robotic teleultrasound diagnostic system to care centers (10), intensive care units (11), and COVID-19 pandemics (12). However, to our best knowledge, none of the applications of the robotic teleultrasound diagnostic system in health check-ups have been reported to date. In this study, we aimed to prospectively assess the feasibility and satisfaction of health check-ups with a 5G-based robotic teleultrasound diagnostic system.

## Materials and methods

### Subjects

This prospective bi-center study was carried out in Hospital #1 (Wuhan Tongji Hospital) and Hospital #2 (China Resources & Wisco General Hospital) synchronously from April to August 2022. The inclusion criteria were volunteers from two corporations in Wuhan, China who agreed to participate in the teleultrasound check-up. The exclusion criteria were those who refused to provide consent and participate in the teleultrasound check-up, and poor, frequently interrupted network signals during the checkup. The study was approved by the institutional research ethics committee of both hospitals, and written informed consent was obtained from each subject.

### Instruments

**Figure 1:**
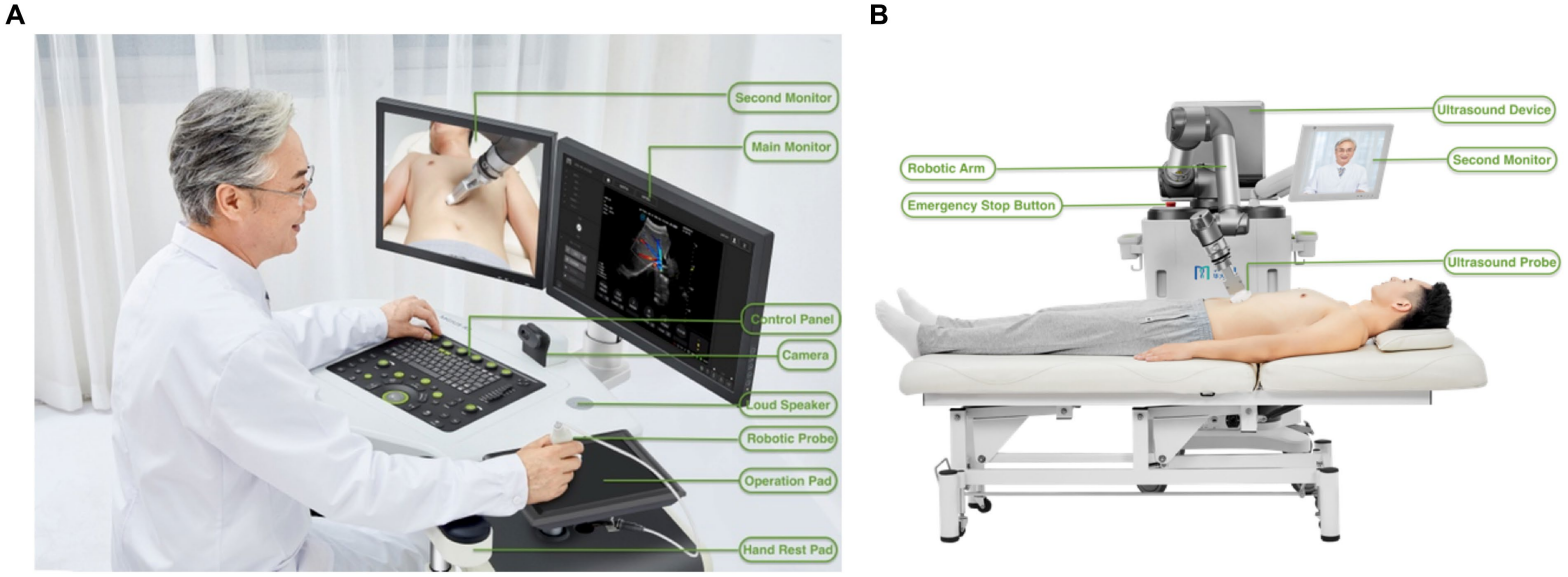
Structural compositions of the robot-assisted teleultrasound diagnostic system. **(A)** Basic components from the right-front view of the doctor-end system. **(B)** Basic components from the rear view of the patient-end system. (We acknowledged the pictures provided by MGI Tech Co.)

The teleultrasound robot system used in this study was MGIUS-R3 (MGI Tech Co., Ltd., Shenzhen, China), equipped with a C5-1 abdominal probe with a frequency of 2.5–5.0 MHz. This telerobotic ultrasound diagnostic device is composed of two parts: a doctor-end system and a patient-end system (Figure 1). The doctor-end system was located in two hospitals where the 5G-based telerobotic ultrasound check-ups were performed by sonologists remotely, and the patient-end system was in two corporations.

The doctor-end system consists of mechanical components, an operation control system, an audiovisual system, and doctor-end control software. The mechanical components are the operation table which acquires operation guidance from the doctor and transfers feedback from the patient-end system. The components of the operation control system include a robotic probe, operation pad, ultrasound control panel, and main monitor. The telerobotic ultrasound mock probe can acquire the positions and postures of the operation methods on the doctor’s side. The operation pad receives the positions and contact forces of the robotic arm on the patient’s side. The combined application of the robotic mock probe and operation pad enables the consistency between the doctor’s and robotic arm’s motion via a series of predefined coordinate system conversions (13). The ultrasound control panel is used for adjusting the various ultrasound parameters of the equipment including frequency, gain, focus, depth, dynamic range, etc. on the patient side to obtain real-time ultrasound images. The main monitor equipped with a high-fidelity 1080P medical-grade screen displays real-time ultrasound images and relevant information. The audiovisual system includes a camera and a second monitor that obtains audio and video signals on the doctor’s side, to help the interaction between sonologists, patients, and assistants synchronously.

The patient-end system consists of mechanical components, an operation execution system, an ultrasound device, an audiovisual system, and patient-end control software. In the patient-end system, the mechanical components receive the doctor’s operation guidance to transfer feedback to the doctor-end system. The operation execution system refers to the robotic arm where an ultrasound probe is fixed at the end of it to perform an ultrasound scan. The robotic arm possesses a high-precision, flexible, and controllable six-dimensional force sensor to access real-time force feedback information during the interaction between the ultrasound probe and the human soft tissue surface. The ultrasound system consists of an ultrasound device designed to acquire, process, and transfer ultrasound images, and an ultrasound probe used for acquiring ultrasound images. The audiovisual system was the same as the doctor-end system, obtaining audio and video signals on the patient’s side to facilitate communication between sonologists, patients, and assistants synchronously. The patient-end control software is applied to acquire ultrasound images on the patient’s side.

Referring to the safety protection issue, the accurate force control sensor and force protection algorithm of the robotic arm can ensure the speed and pressure within the preset maximum limit, such as the vertical and horizontal protection force range of 3–40 N and 0–20 N, respectively in the abdominal examination. If excessive force occurs, the emergency stop button will be triggered and the mechanical arm will stop instantly to fully ensure the patient’s safety.

The successful implementation of the robot-assisted teleultrasound check-up is heavily dependent on 5G networks with the characteristics of high speed, low latency, and large bandwidth (14, 15). The network we used in the study had an average download speed of 240 Mbps and an average upload speed of 45 Mbps. The delay in the process of examination was <250 ms.

### Study design

In this study, the teleultrasound check-ups were performed for volunteers from Corporation #1 located 40 km and 21 km away from Hospital #1 and Hospital #2, respectively, and we also performed the teleultrasound check-ups for volunteers from Corporation #2 located 29 km and 28 km away from Hospital #1 and Hospital #2, respectively. The two corporations were referred to as Corporation #1 and Corporation #2 to protect volunteers’ privacy. All the volunteers were randomly assigned to the two hospitals. Two sonologists (8 and 20 years of experience in ultrasound, respectively) from both hospitals performed all the remote robotic ultrasound check-ups and underwent teleultrasound robot manipulation training in advance.

Before the examination, the on-site assistant registered the subject information and attached the abdominal ultrasound transducer to the probe clamp of the robotic arm. The subject lay on the examination bed in the supine position, with the abdomen exposed. The sonologist adjusted the examination mode to abdominal scanning and started the robotic arm. The on-site assistant applied sufficient ultrasonic coupling agents to the upper abdomen. Then, the sonologist held the mock ultrasound probe to control the robotic arm of the patient’s side to perform scanning. Each male individual underwent ultrasound check-ups of the liver, gallbladder, pancreas, spleen, kidneys, bladder, and prostate. While each female individual underwent ultrasound check-ups of the liver, gallbladder, pancreas, spleen, kidneys, bladder, uterus, and ovaries. In the process of scanning, the sonologist can switch the video button to observe either the subject and surroundings or the orientation and position of the ultrasound probe. Through the high-quality audiovisual communication systems equipped both on the doctor’s and patient’s side, the sonologist can guide the subjects in adjusting their positions and guide the on-site assistant in adding ultrasonic coupling agents. When the ultrasound examination was complete, the sonologist immediately issued the ultrasound report of examination results uploaded to the WeChat mini program from which employees can look over.

The duration of each teleultrasound examination was recorded from the time the first image was stored to the time the last image was stored. After finishing the telerobotic ultrasound examination, the subjects voluntarily selected to fill out the satisfaction questionnaire, including their overall satisfaction degree with the teleultrasound check-up project, the degree of abdominal and urogenital examination comfort, the satisfaction degree of communicating with the sonologist smoothly during this examination, and the satisfaction degree with the sonologist’s professionalism and service. Another question was about the degree of being afraid of the robot arm during the examination (no fear, fear, extreme fear).

### Statistical analysis

SPSS software (version 27.0) was used to perform the statistical analyses. Continuous variables are expressed as mean ± standard deviation and range (normal distribution) or median and interquartile range (non-normal distribution). Categorical variables are presented as counts and percentages. Mann–Whitney U test was used to compare the examination duration between men and women. *p* < 0.05 was considered statistically significant.

## Results

**Figure 2:**
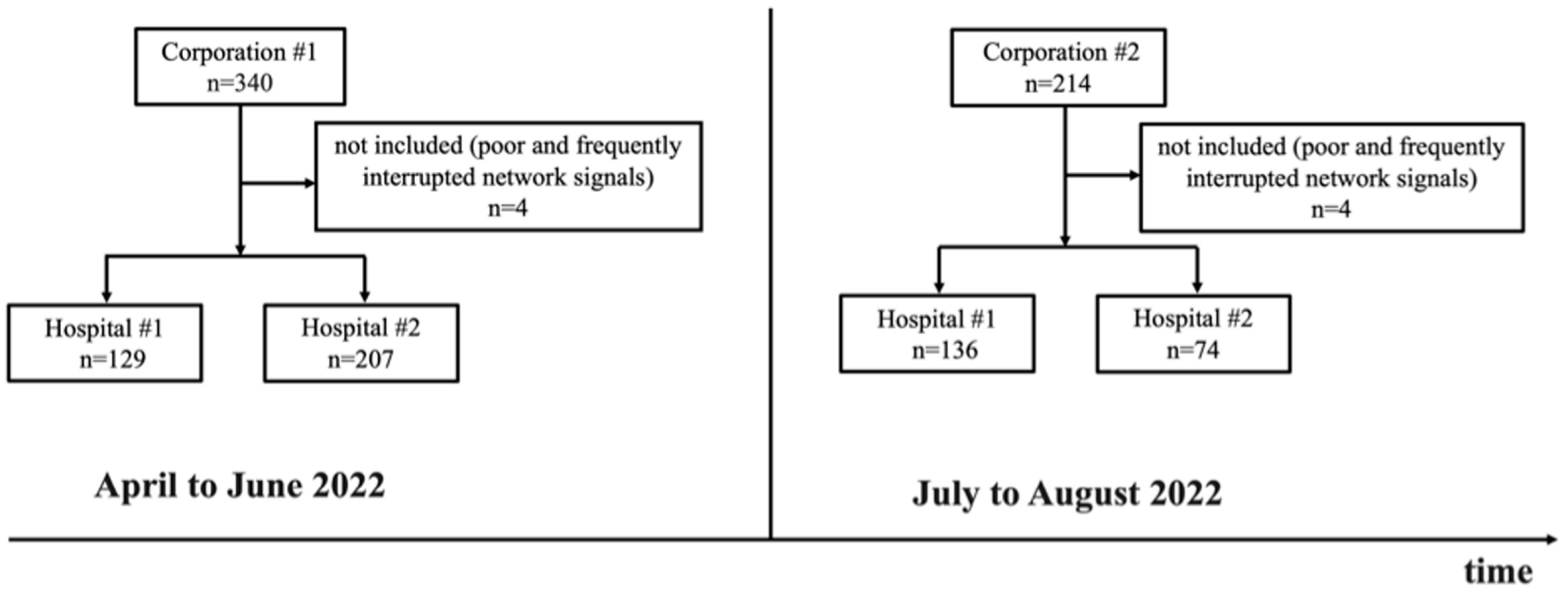
The flow chart of the telerobotic ultrasound check-ups performed for staff from Corporation #1 and Corporation #2 in Hospital #1 and Hospital #2 from April to August 2022.

A total of 554 telerobotic ultrasound check-ups were performed in this study, among which 8 subjects were excluded because of poor and frequently interrupted network signals. Finally, there were 546 subjects (350 men, 196 women; mean age: 35.1 ± 10.3 [range: 17–71] years) included in our study. Thereinto 129 and 207 subjects from Corporation #1 were randomly assigned to Hospital #1 and Hospital #2, respectively from April to June 2022. From July to August 2022 the number of subjects from Corporation #2 randomly assigned to Hospital #1 and Hospital #2 was 136 and 74, respectively (Figure 2).

Among abdominal examinations, 8% of all the subjects abandoned the examination of the gallbladder because of no fasting (*n* = 44). The assessment of the pancreas was limited because of increased body habitus (*n* = 10) and gastrointestinal gas (*n* = 40). Among pelvic examinations, 16% of all the subjects abandoned the examination of the bladder because of not having a well-filled bladder (*n* = 90). The assessment of the prostate was limited because of a poorly filled bladder (*n* = 20). The assessments of 36% of the uterus and 29% of the unilateral or bilateral ovaries were limited due to the poorly filled bladder (*n* = 70) and bowel gas (*n* = 58), respectively. In the rest of the adequately examined organs, 62% (341/546) had abdominal disorders, 14% (75/546) had urinary problems (bladder and kidney), 28% (97/350) of men and 16% (31/196) of women had reproductive illnesses. There was a total of 32 kinds of abnormal ultrasound manifestations found, of which the most frequently diagnosed were as follows: fatty liver, 37% (204/546); prostatic calcification, 15% (54/350); enlarged prostate with or without calcification, 12% (42/350); hepatic cyst, 6% (32/546) (Table 1).

**Table 1:** Clinical characteristics and abnormal ultrasound findings of the included subjects examined at Tongji hospital and China resources & wisco general hospital.

Clinical characteristics and abnormal findings	Tongji hospital, *n*	China resources & wisco general hospital, *n*	Total, *n*
Clinical characteristics			
*Sex*			
Male	169	181	350
female	96	100	196
*Age, y*			
<20	2	0	2
20–40	192	230	422
>40	71	51	122
*Previous surgical history*			
Cholecystectomy	2	5	7
Partial splenectomy	0	1	1
Partial or total hysterectomy	2	0	2
Bilateral salpingectomy	1	0	1
*Abnormal findings*			
*Liver*			
Fatty liver	121	83	204
Hepatic schistosomiasis	0	1	1
Hepatic cyst	15	17	32
Hepatic hemangioma	7	12	19
Intrahepatic hypoechoic nodule	2	0	2
Intrahepatic calcification	19	9	28
Hepatic calculus	1	2	3
*Gallbladder*			
Gallbladder wall thickening	2	0	2
Gallstone	9	4	13
Gallbladder polyps	13	11	24
Cholesterol crystals in gallbladder wall	4	0	4
*Pancreas*			
Pancreatic fat infiltration	2	0	2
Pancreatic cyst	2	0	2
*Spleen*			
Splenomegaly	2	3	5
*Kidney*			
Renal dysplasia	0	1	1
Renal cyst	15	13	28
Renal hamartoma	1	4	5
Calcification of renal parenchyma	1	1	2
Polycystic kidney	1	0	1
Parapelvic cyst	2	1	3
Hydronephrosis	1	0	1
Renal calculus	6	24	30
*Bladder*			
Bladder deposits	3	1	4
*Prostate*			
Enlarged prostate with or without calcification	28	14	42
Prostatic calcification	30	24	54
Prostatic cyst	1	0	1
*Uterus*			
Hysteromyoma	10	2	12
Myometrial strong echogenic foci	2	0	2
Intrauterine device	7	3	10
*Ovary*			
Ovarian cyst	3	3	6
Strong echogenic foci in adnexal area	1	0	1
Pelvic effusion	3	5	8

**Figure 3:**
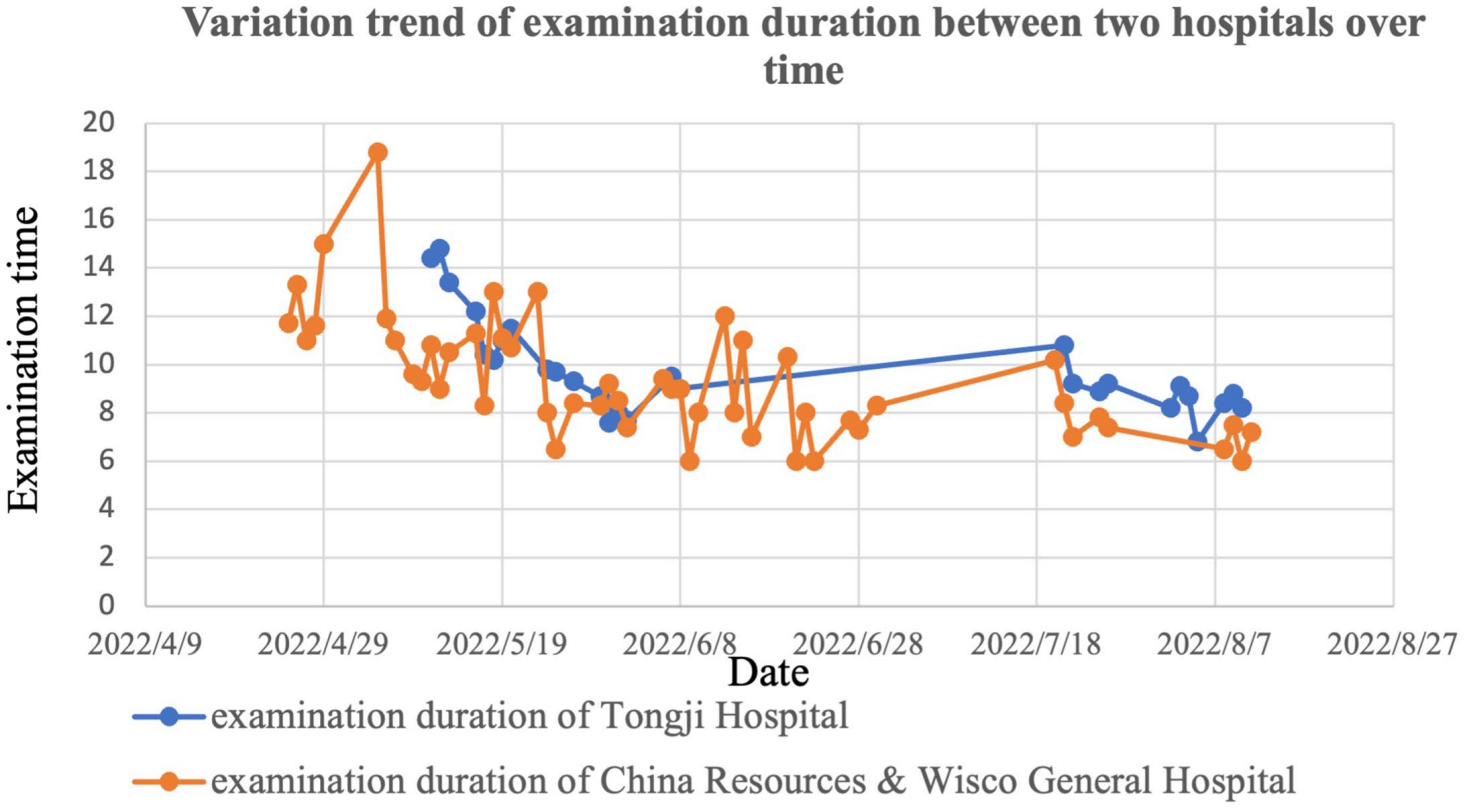
The variation trend diagram of examination duration of Hospital #1 and Hospital #2 from April to August 2022.

The median teleultrasound examination duration (interquartile range) for men was 9 (9–11) min, the longest duration was 22 min, and the shortest was 5 min. While the median teleultrasound examination duration (interquartile range) for women was 9 (7–11) min, the longest duration was 23 min, and the shortest was 5 min. There was no statistical significance of the examination duration between men and women (*p* = 0.236). With the improvement of experience and proficiency, the examination duration showed a downward trend despite the fluctuations for both two sonologists (Figure 3).

A total of 115 subjects responded to the satisfaction questionnaire survey. None of the subjects were dissatisfied with the teleultrasound check-up project. All subjects and 98% felt comfortable with the abdominal and urogenital examination, respectively, while 2 subjects were dissatisfied with the urogenital examination. One hundred and fourteen subjects felt easy communicating with the sonologist using the audio-video system remotely, whereas only one subject felt difficulty in communicating with the sonologist. Ninety-nine percent of the subjects were satisfied or very satisfied with the sonologist’s professionalism and service except one (Table 2). The percentage of no fear, fear, and extreme fear of the robotic arm during the examination was 96, 2, and 2%, respectively.

**Table 2:** The survey results of the satisfaction questionnaire from 115 volunteers.

Questions	Strongly agree, *n* (%)	Agree, *n* (%)	Disagree, *n* (%)
Q1 Are you satisfied with the 5G-based robotic teleultrasound check-ups?	70 (61)	45 (39)	0 (0)
Q2 Do you feel comfortable during the abdominal examination?	79 (69)	36 (31)	0 (0)
Q3 Do you feel comfortable during the urogenital examination?	86 (75)	27 (23)	2 (2)
Q4 Can you communicate with the doctor smoothly during the examination?	74 (64)	40 (35)	1 (1)
Q5 Are you satisfied with the doctor’s professionalism and service?	80 (70)	34 (29)	1 (1)

Values are *n* (%).

## Discussion

In this prospective bi-center study, 546 teleultrasound check-ups were successfully completed using a 5G-based telerobotic ultrasound diagnostic system. Based on the assessment of examination results, examination duration, and satisfaction questionnaire survey results, the findings in our study demonstrated that a 5G-based robotic teleultrasound diagnostic system has a considerably high level of feasibility and satisfaction in health check-ups and could serve as a unique solution for the application of teleultrasound check-ups in underserved rural and/or remote areas lacking professional sonologists and medical resources.

A previous study reported that ultrasound-assisted health check-ups as part of a regular health screening visit could identify more clinically meaningful abnormalities than a traditional exam in older adult patients (16). Recently a study by Rosborough et al. found that ultrasound health check-ups could identify frequent abnormalities and new important chronic conditions in wellness patients 65–85 years old and elucidated that the evaluated benefits of these abnormalities were rarely negative and always mild to moderately positive (3). Nevertheless, in China, hospitals, especially tertiary hospitals, harbor the most quality medical resources while primary care institutions are lacking professional sonologists and ultrasound devices (17). The significant regional disparities in the geographic distribution of medical resources cause barriers for people living in underserved rural and/or remote zones access to routine ultrasound check-ups. Telemedicine, therefore, as a well-suited tool, attempts to reduce geographic barriers to expand the scope of access to healthcare and medical services (18). Telerobotic ultrasound, as another kind of telemedicine, may be an important method for minimizing the distance from sonologists and ultrasound devices to ensure equitable access to medical services for both urban and rural or remote individuals. A previous study demonstrated the feasibility of using a teleultrasound robotic diagnostic system (MELODY system, France) to build a service delivery model to provide ultrasound services in rural and remote groups (19). Whereas our study provided a unique solution for populations in low-resource and underserved settings to have regular ultrasound check-ups using a teleultrasound robotic diagnostic system (MGIUS-R3) in a real-time, or synchronous manner.

In our study, among all the abnormal ultrasound manifestations, abdominal disorders accounted for the most, of which fatty liver was the most frequently diagnosed, which was consistent with the findings of Huihui Chai et al. (10). Fatty liver is a highly prevalent disease, leading to liver fibrosis and cirrhosis in a gradual process (20). Abdominal ultrasound is the first-line method to screen and monitor fatty liver disease (21). When screened and diagnosed as having fatty liver, one should make modifications to lifestyle or undergo pharmaceutical treatment (22). However, a large number of individuals residing in low-source or underserved locations are undiagnosed due to the difficulty in accessing regular ultrasound screening. This study demonstrates that a 5G-based telerobotic ultrasound diagnostic system may be a working-well alternative to traditional ultrasound examination for routinely screening the fatty liver.

Previous studies reported that the examination duration of the telerobotic ultrasound was longer than the traditional ultrasound (10, 23). Our experience was the same although our study does not compare the examination duration of the telerobotic ultrasound to the traditional ultrasound. This longer examination duration may be correlated with the subjects changing their positions frequently when examined in different parts of their body, the need for on-site assistants, and the time spent manually adjusting the original position of the probe after the probe was activated (23). Nevertheless, the use of telerobotic ultrasound diagnostic systems in ultrasound check-ups reduced the total time cost in comparison with conventional ultrasound (24). For example, healthy individuals have no need to travel long distances to reach a nearby hospital and undergo an ultrasound scan. In addition, for each of the two sonologists, the examination duration showed a downward trend despite the fluctuations. The reason may be that sonologists’ experience and proficiency improved as time goes on (13).

With respect to the satisfaction questionnaire surveys, all the subjects were very satisfied or satisfied with the teleultrasound check-up project which indicated that the 5G-based telerobotic ultrasound diagnostic system could be satisfactory in ultrasound check-ups. Prior studies have also made the same conclusions (19, 25). Whereas two participants were dissatisfied with the urogenital examination. One reported that the reason was that on-site assistants applied ultrasonic coupling agents repeatedly because of their uneven distribution which wasted much time, and another reported that an unstable network with interruption accidentally disturbed the examination process and required a restart of the robotic arm. Actually, it was rather hard to make the on-site assistant being the same person due to the manpower shortage and difficulty in personnel coordination, and the new on-site assistant needed to be trained and skilled again. Theoretically, our network based on 5G could ensure fast and smooth simultaneous transmission (26). However, network malfunctions still occurred occasionally (27). Our solution was to optimize the network such as increasing the bandwidth from 10 M to 15 M of the server. In addition, one subject felt difficulty communicating with the sonologist as he cannot hear the sonologist’s voice clearly, making him communicate with the sonologist repeatedly, which was a waste of time. The possible cause may be that the speaker went wrong on the patient’s end. In the following work, this problem could be solved by optimizing the control algorithm or replacing the speaker (28). Although 96% of the subjects showed no fear of the robotic arm during the examination, 4% were still more or less afraid of the probe held by the robotic arm. The probable reason was that they have not gotten used to this new type of check-up yet. As Adams et al. reported that there were several patients using the word “did not seem real” or “weird” to express their apprehension with this teleultrasound robotic technology compared with traditional ultrasound examinations (19). Another advantage worth mentioning of the robot-assisted teleultrasound check-up was that a hand rest pad was designed near the operation pad, supporting the sonologist’s elbow during the ultrasound scanning which is helpful for reducing the arm pressure of operators working for a long time, and the incidence of occupational diseases of sonologists (27, 29). In addition, a single sonologist could operate a teleultrasound system on multiple patients’ ends, providing the opportunity to reach more rural and/or remote areas and facilitating the rational allocation of high-quality medical resources.

However, there are also some limitations in this study. First, our subjects were urban volunteers rather than people from rural and/or remote areas and we hypothesized that our study could be applied to underserved rural and/or remote areas. Second, we only performed the teleultrasound check-ups for subjects void of comparing the diagnostic accuracy of it to the conventional ultrasound as previous studies found excellent agreement between conventional ultrasound and telerobotic ultrasound (23, 30, 31). Third, the operating angle of the mechanical arm has restrictions in some directions forbidding the sonologist from reaching the locations where they want to examine.

In conclusion, the 5G-based teleultrasound robotic diagnostic system in health check-ups is feasible and satisfactory, indicating that this teleultrasound robotic system may have significant application value in an approach that minimizes transfers and reduce barriers to achieving health resources and services for people in underserved rural and/or remote areas.

## Data availability statement

The data analyzed in this study is subject to the following licenses/restrictions: The datasets presented in this article are not readily available because of privacy and ethical restrictions. Requests to access the datasets should be directed to the corresponding author. Requests to access these datasets should be directed to X-WC cuixinwu@live.cn.

## Ethics statement

The studies involving human participants were reviewed and approved by Tongji Medical College of Huazhong University of Science and Technology and China Resources & Wisco General Hospital. Written informed consent to participate in this study was provided by the participants’ legal guardian/next of kin. Written informed consent was obtained from the individual(s), and minor(s)’ legal guardian/next of kin, for the publication of any potentially identifiable images or data included in this article.

## Author contributions

X-WC and H-RY designed the study. J-YR, Y-ML, and B-SL gathered and analyzed the data. J-YR drafted the first version of the manuscript which was then discussed and revised critically by J-YR, Y-ML, B-SL, Y-XP, X-FP, H-RY, and X-WC. All authors contributed to the article and approved the submitted version.

## Funding

This study was funded by the National Natural Science Foundation of China (no. 82071953), the Ministry of Science and Technology of China (no. G2022154032L), and the research project of the Wuhan Municipal Health Commission (no. WG19C01).

## Conflict of interest

The authors declare that the research was conducted in the absence of any commercial or financial relationships that could be construed as a potential conflict of interest.

## Publisher’s note

All claims expressed in this article are solely those of the authors and do not necessarily represent those of their affiliated organizations, or those of the publisher, the editors and the reviewers. Any product that may be evaluated in this article, or claim that may be made by its manufacturer, is not guaranteed or endorsed by the publisher.
